# Parental substance and alcohol abuse: Two ethical frameworks to assess whether and how intervention is appropriate

**DOI:** 10.1111/bioe.12920

**Published:** 2021-07-10

**Authors:** Anke Snoek, Dorothee Horstkötter

**Affiliations:** ^1^ Department of Health, Ethics and Society MHeNs, School for Mental Health and Neuroscience at Maastricht University The Netherlands

**Keywords:** autonomy, beneficence, child abuse, ethics of care, moral dilemmas, normative frameworks, parenting, substance dependency

## Abstract

Ethical frameworks can support professionals’ decision‐making. Here, we identify two ethical frameworks to analyse the best support for families that struggle with parental substance or alcohol abuse. The first framework, which we call ‘the framework of conflicting interests’, is most prominent in the literature. Here, the interests of parents and children are weighed against each other using the medical ethical principles of respect for autonomy, justice, beneficence, and non‐maleficence. The second framework is most prominent in a series of interviews we conducted with alcohol‐dependent parents and professionals working in addiction care and youth care. This framework aligns more with an ethics of care, and starts with the assumption that the interests of people who are close to each other are often intertwined. This framework does not so much look at conflicting interests, but at relationships and vulnerability. We label this the ethics of care framework. In this article, we show the value of both frameworks and how they can support ethical decision‐making.

## 1 INTRODUCTION

Children of parents with substance dependency often report a lower sense of wellbeing later in life compared with their peers with non‐dependent parents.^1^ However, the care for these children and their families is often complicated. Firstly, professionals and lay people who come into contact with these families are unsure when to intervene, as interfering with peoples’ personal lives brings about moral dilemmas.^2^ This leads to a reluctance to act among professionals and lay people.^3^

Secondly, treatment is complicated by a lack of integrated care, as substance‐orientated and child‐orientated services are distinct.^4^ Adults with substance dependency are supported by alcohol and other drug (AOD) workers, while their children are supported by child welfare services (CWS). Unfortunately, AOD workers often find it hard to ask their clients about family life, leaving it unclear what support the children need, while CWS professionals often describe parents as unwilling to openly discuss their substance‐use problems with them. The collaboration between services is further complicated by differing financial flows, uncertainty about what information can be shared, different treatment goals,^5^ and divergent values between the services.^6^

Some suggest that family care can be improved by making the implicit values of professionals explicit:^7^ ‘Successful collaborative models appear to rely heavily on a foundation of values clarification and creation of a shared philosophy’.^8^ Furthermore, ethical frameworks can support decision‐making.^9^

Our aim is to identify the normative frameworks that parents and professionals use when talking about dilemmas in care. We first outline our method, and then describe two frameworks that we identified in our literature search and our interviews: the framework of weighing conflicting interests, and the ethics of care framework. We discuss the strengths and limitations of the two frameworks, and demonstrate how they can support both professionals and lay people. We argue that an awareness of these frameworks can help collaboration between services, and collaboration between treatment‐providers and families.

## 2 METHOD

To identify the normative frameworks currently present in interventions for families struggling with parental substance abuse, we (1) conducted a literature search, using keywords for parental substance dependency and ‘ethical’, ‘moral’, ‘dilemma’; (2) held interviews with alcohol‐dependent parents; and (3) organized focus groups with professionals working with these families.

We interviewed, in the Netherlands, 10 alcohol‐dependent parents and held three focus groups with professionals: two with CWS workers (*n* = 5), and one with AOD workers (*n* = 6). Practical hurdles, especially the high workload in child welfare and AOD services, prevented us from doing more interviews. However, the interviews that were conducted contain very rich data that allowed us to identify important themes emerging from the interviews. While more interviews might have added to the data, we do not think that they would have added further themes. For this reason, we consider our material to be saturated. We focus specifically on alcohol dependency because this is the prevalent type of substance misuse for parents, and studies have indicated that it is under‐detected and its impact underestimated.^10^ There was very little literature focusing on ethical dilemmas with alcohol‐dependent parents, so we broadened our search to substance dependency in general.

Hour‐long interviews with parents were conducted individually at their homes or within the care facility, depending on the respondent’s preference. We combined semi‐structured questionnaires with a timeline narrative approach.^11^ Focus groups with professionals took place within the AOD services and CWS. These semi‐structured interviews, lasting about one hour, were recorded, fully transcribed verbatim, and analysed in NVivo. Both researchers analysed the data independently, to ensure inter‐rater reliability. Given the comparatively low *N*, inter‐rater reliability was established in a dialogical way between the two authors.

When integrating moral theory and empirical research, one can chose a bottom‐up, top‐down, or dialogical approach. We chose a dialogical approach,^12^ adopting a strongly interactive process alternating between current ethical literature and the views and experiences of our participants.

## 3 RESULTS

Because of the interactive and iterative analysis method, theoretical and empirical results are closely interwoven in the description of our results.^13^

The ethical literature and interview material reveals that two largely distinct ethical frameworks play a role in determining support for families. The first, ‘the framework of weighing conflicting interests’, was most prominent in the literature. Parents’ and children’s interests are weighed against each other using the medical ethical principles of respect for autonomy, justice, beneficence, and non‐maleficence. The second, ‘ethics of care’, framework was most prominent in the interviews, highlighting that the interests of people who are close to each other are often intertwined rather than distinct. Relationships and vulnerability are key foci. Remarkably, when searching for ‘ethics’ and ‘moral’ in child welfare and addiction literature, the ‘ethics of care’ framework did not come up. We now present the two frameworks and discuss their respective values and limitations.

### 3.1 The framework of weighing conflicting interests

The literature on moral and ethical problems in the care of families with parental substance abuse often uses a language of conflicting interests between parents and children, and a normative framework is invoked that focuses on a proportionate weighing of these interests.^14^ The term ‘interests’ is thereby used in a rather broad sense, covering not only direct interests, but also people’s personal needs as well as their rights. However, given that it is also in people’s interest that their needs are fulfilled and their rights respected, we will invoke only the more general term of ‘interests’, which subsumes the needs, rights and other topics that a person may have an interest in realizing.

The framework first identifies these different interests. Beauchamp and Childress' four ethical principles are often used: respect for autonomy, non‐maleficence, beneficence, and justice.^15^ Next, these interests are weighed against each other. Under which circumstances can we overrule parental autonomy in favour of the child’s beneficence? This framework is most strongly articulated in determining care for pregnant substance users and their fetuses,^16^ and in literature on child abuse in general.^17^

Dondorp and De Wert define the dilemma in care for pregnant substance‐dependent women as a conflict between the maternal right to self‐determination and the fetal right to non‐harm.^18^ Since the woman’s right to self‐determination is ethically and judicially protected, overriding that principle and invoking pressure or coercion to prevent harmful drug use requires justification. However, protecting the fetus is not sufficient justification. The intervention should also meet the criteria of effectiveness, proportionality, and subsidiarity. It must be able to prevent the harm, it should not be more invasive than necessary, and there should be no alternative that is less invasive and as effective. The goal *and* the means must be ethically justifiable. This shows that coercive means can be justifiable, but only given preconditions: whether the harm is serious enough and can effectively be prevented, and whether less invasive means are available.

Lambert, Scheiner and Campbell also describe competing interests: ‘Maternal–fetal conflict arises when a pregnant woman’s interests as she defines them (in this case drug use or abuse) conflicts with the interests of her fetus: to be born healthy with the best possible chance of meaningful survival’.^19^ How can the competing interests of maternal autonomy and fetal beneficence be balanced? The authors suggest that maternal autonomy is already compromised by addiction, so can be overruled. However, they conclude that justice should be decisive. They consider the current drug laws to be unjust, because they target some groups more than others. The same behaviour is blamed more in pregnant women than in fathers; poor people and people of colour suffer more from anti‐drug laws than white, upper‐class people. Legal punishments for using crack cocaine (used by poor people and by people of colour) are much higher than those for using regular cocaine (used by white, upper‐class people).^20^ So an ethically justifiable approach does not focus mostly on the fetus’ benefit, but is just, meaning that it provides good healthcare and better drug treatments with less stigma towards vulnerable groups: ‘It is the responsibility of the health care system and lay communities to advocate for the ethical, moral and just treatment of pregnant women and their children, including women with histories of perinatal substance use or dependency’.^21^ This will improve the situation of pregnant women and thereby also benefit the fetus.

A second issue is the reluctance of professionals to report their concerns about families to child‐protection agencies. Owing to the lack of literature, we included the ethical justifiability of mandatory reporting in our search. Here, the weighing conflicting interests framework is also prominent. The interests of professionals are weighed against those of the children or parents they encounter. A total of 40% to 70% of all cases identified by healthcare professionals go unreported.^22^ Personal inhibition—ideas like ‘children belong to their parents’ or mistrust in the child protection services—is the main reason why healthcare professionals do not report. Greipp uses the four principles to outline how the professional responsibility of nurses should overrule their inhibition: ‘To ignore a suspected situation of abuse would be a violation of beneficence and nonmaleficence. It certainly would violate the principle of justice, because no human being deserves to be abused’.^23^ Remarkably, she does not specify how the principle of autonomy relates to nurses’ reluctance. Greipp weighs the principles against personal inhibition. Kalichmann describes mandatory reporting as an ethical dilemma for professionals.^24^ Although they have a legal obligation to report, they are also bound by confidentiality. Respecting confidentiality is a way to maintain the therapeutic relationship, offer the parent treatment, and improve the children’s lives. Breaching confidentiality can harm trust. However, Kalichman argues that professionals might mistakenly associate respecting confidentiality with winning trust. Parents could also interpret this behaviour as lack of care. If it is communicated to the parents in the right way, reporting could also show that a professional takes the family’s struggle seriously.^25^ By making explicit what confidentiality and trust mean, it becomes clear how interests can conflict but also merge.

Feng and colleagues describe how practitioners struggle with conflicting principles.^26^ Beneficence, non‐maleficence and justice are opposed to the principle of respect for parental autonomy. The framework of conflicting interests can help in an analysis of the tension, but is not able to provide a solution because it cannot explain which value or principle should be prioritized. Moreover, Feng and colleagues conclude that there is no clear understanding of what beneficence or non‐maleficence mean and require in this context: for some children, it might mean protecting them from their parents, while others might suffer from separation, stigmatization and their stay in a children’s home.

These are some examples to show that an ethical framework based on medical ethical principles and the identification and balancing of different interests of different parties dominates the current ethical discourse. This framework helps justified decisions to be made in the hardest cases. However, it entails the inherent risk of presenting the rights and needs of parents and children as conflicting, and thereby of pitting the two groups against each other.^27^ A framework of conflicting interests might have the consequence of further weakening already vulnerable families, eroding parents’ trust in professionals, and encouraging punitive approaches towards parents rather than restoring bonds.^28^ In a framework that weighs interests, parents are presented as a potential threat to their child and to society, and the bond between children and their parents is easily overruled for the sake of their safety.^29^ The principle of beneficence could be misused: government officials may ‘overstep the limits of appropriate intervention into private matters’ when they ‘act in the name of beneficence’.^30^

### 3.2 The ethics of care framework: focus on vulnerability and relationships

Concerned parents themselves used quite different language from ‘conflicting interests’ when talking about parenthood and addiction. They did not mention how their interests conflicted with their children’s; nor did they contrast their autonomy with their children’s wellbeing. Instead, they expressed their struggles to care for their children, to maintain good relationships and protect them.

### 3.3 Experiences of parents

In their narratives around parenthood and substance dependency, two themes stood out: substance use as a means to cope with adverse life events; and worries for the wellbeing of their children. The parents described the circumstances that led to their drinking problem, namely what went wrong and how they had to cope with trauma, violence, and poverty. Drinking was initially a way to cope, but eventually became an additional problem. No parent described their drinking as an autonomous lifestyle choice. Parents described their children as the most important things in their lives. Within their adverse circumstances, they did their best to take care of their children, and to protect them from their stress. They worried extensively about the children’s wellbeing and stated that they hoped their children would have a better life than they had. They sometimes expressed anger at caregivers, because they had not paid enough attention to the needs of their children, or because they did not take them seriously as parents, ignoring their parental knowledge of their children’s needs. They also were proud of some aspects of their parenting in these hard circumstances, for example children’s academic achievements, managing to pay for the children’s sports, and their children’s positive character traits, such as kindness. They acknowledged that their drinking might have harmed their children and deeply regretted this.

Parents described their wellbeing and that of their children as intertwined. One parent described how, regardless of her alcohol problem, she always managed to get her children in clean clothes and provide lunchboxes for school. She states:

And that is my luck, that I have strong values from my upbringing, that basically I managed to do quite fine. Despite my addiction, I took my responsibilities. But I did not take good care of myself. And you know, if mothers are unhappy, it is hard for them to make their children happy, because the children feel it. (Monique, children aged 8, 11, 14.)

Although their children’s happiness has always been their priority, parents noticed that if they had low wellbeing, their children struggled as well. On the other hand, parents described how their children’s struggles hugely affected them as well. One parent described how her daughter struggled with depression, and how she worried whether her daughter would get the right support from her foster parents. One mother said:

I worried a lot about whether they would blame themselves. Whether they would think it was their fault that I was drinking. If they just had normal naughtiness, and I would get angry at them, whether they would think that this would cause my drinking. (Barbara, children aged 8 and 6.)

Professionals described parents as care‐avoiding regarding their drinking problems. This view of parents was also dominant in the balancing conflicting interests framework. In the literature, parents are often portrayed as defending their autonomy and privacy, yet parents described themselves as wanting help for the *underlying problems that caused their drinking*, and support for their children. Asked about this apparent discrepancy, parents denied avoiding care. Rather, they felt that no one was interested in helping. Several parents described noticing that others were aware of their problems, but did nothing.

The school knew I was drinking. The police had been at our door so many times [because of domestic violence committed by her husband], but they never did anything to help my children. (…) I wish they had intervened earlier. (Monique, children aged 8, 11, 14.)

Drinking apparently changed me, because people in the neighbourhood started to eye me, but no one ever said something. (Steve, child aged 18.)

This lack of interference was not interpreted as respect for their autonomy or private life; rather, it reinforced feelings of hopelessness and worthlessness. When asked whether they would have been open to support, parents admitted that probably that would not have been easy. They felt especially vulnerable when help included criticism of their parenting or drinking.

Don’t threaten my children, or you will get into a fight with me. And that is the ambivalent part: on the one hand, you are hoping so hard for someone who pressures you to seek help… (Monique, children aged 8, 11, 14.)

Parents felt that it was unjust that others focused on condemning their drinking while so much more was going on that was at least equally important. They longed for support for these deep‐seated problems, but without criticism. Parents suggested that they could best be reached with sincere compassion. This approach would also be the key to their drinking, which they considered a symptom of underlying problems.

Maybe first have a conversation with the parent. Not in an accusing way, like ‘oh, you are an alcoholic’ (…) but: ‘Is there something we can do to help you?’ (…) If the intention is care, and not accusation, that is very important. (…) When you are addicted, you feel immediately criticized as a parent ‘ah, you are not taking good care of your children’, I am very sensitive to that. (Monique, children aged 8, 11, 14.)

If my brothers and my sister, if they would have said: ‘Steve, what is happening with you? Is there something that is worrying you? Are you bottling things up?’ Then maybe I could have shared my feelings surrounding my father’s death, and things might have been different. Then I would have had support earlier. (…) I could have had a more normal life than now. (Steve, child aged 18.)

When criticized as a parent, in line with the language of conflicting interests, they would fight the person suggesting this. However, they longed for sincere compassion. An attitude of care would help them to accept support.

To summarize, parents described a strong bond with their children, and stated that their own wellbeing and their children’s wellbeing were interrelated. Concern for their common wellbeing, an understanding of their vulnerability, and compassion would help them. They also hoped for praise for what they did do for their children in spite of their difficult circumstances. The language used to describe their and their children’s situation is closely linked to the concept of ethics of care. Therefore, we now evaluate the value of an ethics of care normative framework for providing support and interventions to families with parental substance abuse.

### 3.4 Ethics of care

The ethics of care approach argues that the starting point of ethical action should not be universal principles such as autonomy and beneficence, but people’s real‐life situation: their vulnerabilities, needs, and social embeddedness.^31^ Universal principles picture people as autonomous and self‐determining actors; ethics of care questions this picture. Humans are also vulnerable, connected, and dependent on others. The question is not ‘what is justified?’, but ‘how should we respond?’ Ethics of care analyses *values* that are important for care relationships and hence is not based on rights and interests. It further argues that responsibility is distributed and is not placed solely on particular individuals. The responsibility of alcohol‐dependent parents towards their children, is mitigated by how adverse or traumatic experiences foster and uphold parental alcohol abuse. One should not merely hold them responsible for any failures to seek help and support, but also consider how various factors can make this difficult. Social stigma, poverty, lack of affordable childcare while in treatment, and feelings of low self‐esteem can all be barriers to treatment.^32^

So an ethics of care framework focuses on relationships—both between the client and caregiver and between the client and loved ones—and on the social contexts in which people make decisions.

The narratives of the parents we interviewed echoed this ethics of care framework. While from a theoretical point of view universal principles of autonomy and beneficence/non‐maleficence might be relevant for ethically evaluating families’ situations, for most parents this is neither sufficient nor appropriate for evaluating their situation. An ethics of care framework appears to be more appropriate and better able to lead to a course of action.

### 3.5 Experiences of professionals

We also talked to professionals from both addiction care and CWS. Revisiting professionals’ views, it seems that they adhere to both normative frameworks when considering the legitimacy of interventions.

Professionals rarely use the principles of autonomy, beneficence, non‐maleficence and justice, but they made strong use of a language of conflicting interests between parents and children, and between care systems for adults and for their children. When asked about collaboration between services, CWS professionals sometimes considered themselves as opposing those providing addiction care. CWS professionals described how they differ from AOD workers in their views on how parents’ privacy rights should be balanced against support for children.

They [*AOD workers*] are afraid. We ask [*them*]: ‘how is the treatment of this lady going with regard to her alcohol dependency?’ [*They reply*] ‘No, that is the privacy of the client, we won’t discuss that’. (…) But can’t they look at the bigger picture? What this means for the children? No, they refuse to do that. (Ellen, Focus group Child Welfare.)

Sharing information openly. The possibilities have luckily expanded. They [*AOD workers*] are now obliged to ignore privacy when children are involved. (Harold, Focus group Child Welfare.)

Professionals repeatedly expressed a language of ‘choosing sides’:

I think we are far too scared to take a stand for children, against the parents. I think that's what happens a lot. The needs of parents are taken into account far too much. ‘Those poor parents and this and that and we should give them another chance’. But we think: ‘what does it do to children? What does it do to children, if you should never take a friend home with you, because your mother is always drunk’. (Harold, Focus group Child Welfare.)

This morning I had an intake with a woman who wants to be admitted here. She has a child who is one year old. And she asks me ‘I won’t lose my child, right?’ And my first response was to say: ‘No no no, it will be alright’. But then I thought, I shouldn’t say that, because if she is admitted here, and leaves without being discharged, then we will probably report the situation to child welfare. (…) But my first response was ‘don’t worry’. (Babette, Focus group Addiction Care.)

This professional’s first response was to side with the parent and reassure her that her child would not be taken away, only to realize she could not make such a promise. Thus, professionals do consider their role and the evaluations they make in the context of weighing conflicting interests.

However, we also found ethics of care language in the interviews:

Because often it is so polarising, isn’t it? You find yourself positioned opposite of each other. While in fact, you want the same: that the child is thriving. (Ellen, Focus group Child Welfare.)

Yes, that is why there is so much resistance, naturally. Not only because they lose their child, and their relationships with this child, but because they need their children so badly. That is why they are getting worse when they lose their child. And the intervention turns out not to be helpful, because you remove the child from home, so he can regain his breath, while the child only thinks: ‘I have to be with my mother and make sure that she is okay’. So the intervention turns out to be counter‐productive. (Iris, Focus group Child Welfare.)

Here, the child had been removed from home for his own best interest, but he walked a great distance to check whether his mother was okay. AOD workers also acknowledge the value of this interconnectedness.

If you take the parent–child relationship as your focus, if you define that as your client, then you can look both at the problems of the parents, that of the children, and the interaction between both. Then you have it all together, the interests of the children and that of the parents. Especially when the children are still young, it is hard to see them separately from their parent. And you can’t see the parent as separate from them either (…) It’s always a system. Sometimes you have to intervene and protect the interests of the child, but still the parent stays parent, and the child stays their child. That is very important to realize. (John, Focus group Addiction Care.)

Professionals want to treat parents and children together, acknowledging their connectedness. They also experience the language of conflicting interests as polarizing, obscuring the shared interests in children’s wellbeing.

### 3.6 The ethics of care framework is underused in the theoretical literature

In a follow‐up search, in which we explicitly added the key term ‘care ethics’ to our search for ‘substance dependency’ and ‘parenting’, we indeed could identify this ethics of care framework in articles on families struggling with parental substance dependency. Two articles on substance dependency used an ethics of care framework, although labelled slightly differently. Both articles are in the context of perinatal substance use.^33^

Marcellus suggests that instead of abandoning the principles of autonomy and beneficence, we should re‐conceptualize them from a relational and contextual perspective, to comprehend the situation of the women concerned. Autonomy, for example, is often defined as being independent. However, particularly in perinatal situations, the mother and child are not two independent individuals. This prohibits an individualized approach: autonomy and beneficence are inherently relational rather than individual. Mother and child treated as a unity would be better cared for than when imposing some abstract individualized principles on vulnerable families. A relational approach can foster mutual respect, engaged interaction and trust between professionals and families.

Similarly, Söderström and Skolbekken argue that in order to solve ethical dilemmas in pregnancy and substance abuse, the four medical ethical principles should be complemented with a relational ethics approach.^34^ Women find it easier to improve their lives during pregnancy when professionals help them to foster relationships, rather than coercing them to live up to some external standard.

To date, the ethics of care framework has earned its standing in many bioethical fields. However, it seems underused in debates on how to organize care for families with parental addiction. Given that both parents and professionals implicitly consider this approach beneficial, we consider it worthwhile to make this framework explicit in order to strengthen professional decision‐making processes.

### 3.7 Discussion: strengths and limitations of the two frameworks

We have identified two ethical frameworks that can be invoked by professionals when determining ethically good or justified care for families with parental substance abuse. Both frameworks—weighing conflicting interests and ethics of care—have earned their standing in discourses on good care. We now discuss their respective strengths and limitations.

Regarding conflicting interests, both in the theoretical literature and in practice, many find the principles of autonomy, beneficence, non‐maleficence and justice beneficial in analysing conflicts in care. These principles are anchored by law, for example in privacy laws or respect for patient’s autonomy through informed consent.^35^ Moreover, this framework can be useful to identify conflicts experienced by professionals.

The current predominance of the framework of conflicting interests seems understandable. Traditionally, care facilities for addicted parents have been set up separately from care services established for their children. Adults are thus looked after in services providing treatment for addiction, regardless of whether they have children or not. Children are looked after by youth care systems, who have no mandate to treat the parents. The facilities for addiction care on the one hand and youth care on the other have separate infrastructures, financing, treatment goals and interests. With the exception of some clinics that allow addicted parents to be admitted with their young children, care is typically structured separately.^36^ Balancing conflicting interests between parents and children reflects this division of care facilities rather neatly and can help us to understand the tensions that arise between care services for parents and for children.

This framework, however, has serious risks for families who are functioning problematically but where there is still mutual interest and bonding.^37^ In being approached by separate organizations, parents and children risk being pitted against each other^38^ if interests are considered to be conflicting. Only one party (parent or child) is the focus for the provision of care, and little guidance on what should be done is given to professionals.^39^

Parent–child relationships usually have few competing but many shared interests. Parents and children are usually deeply connected, and they value this connection. The parents we interviewed confirmed this. Children also care for the wellbeing of their parents, and not only for their own best interests. This was apparent in the youngster who walked a great distance home to check on his mother.

The language of conflicting interests fails to capture interwoven relationships and the vulnerabilities of the two parties, and does not suggest how to provide good care for the parents and children together.

The ethics of care framework avoids this polarization by not separating roles or interests. Our interviews show that, for parents in particular, such a view makes it easier for them to accept support and acknowledge their struggles. Acknowledging the harsh situations that parents are often in, and giving them credit for their efforts, even if sometimes unsuccessful, can also foster responsibility. An open attitude, rather than threats of child custody or other coercive interventions, might result in a better situation whereby parents take responsibility to seek and accept help rather than avoiding it for themselves and their children.

The basic assumption here is that there is a meaningful interconnectedness between parents and children. This also highlights the limits of this framework. When this bond is very limited or negative, and intentional harm or neglect occurs, the ethics of care framework is not applicable and does not support ethically good decision‐making.

The goal of the ethics of care framework is not to keep children with their families just because it focuses on interconnectedness. The framework does not determine the outcome of the intervention (whether a child stays in the home or not); it determines how parents are addressed, what language is used, and the role of parents in devising solutions.

To illustrate: A Dutch project run by public health institutes is currently using an ethics of care approach to reach vulnerable women in order to encourage them to use contraceptives. There are many unplanned and unwanted pregnancies in this group, and the wellbeing of these unplanned children is a concern. The initiative to support this group is called ‘Pregnant? Not now’, and the project leaders oppose coercive means.^40^

Nurses were trained to start open conversations with the women about their sex life, their current desire to have children, and their knowledge about contraceptives. Other professionals were reluctant to discuss these issues, but nurses found that if they were approached openly and without judgment, many women were willing to discuss these topics. Most of the women had no desire for children, but lacked knowledge about contraceptives. Furthermore, contraceptives were often impractical or unaffordable. These hurdles often required minimal financial and practical support. More importantly, however, when approached compassionately and supportively, rather than with pressure and coercion to prevent pregnancy, up to 86% of the women agreed voluntarily to use contraceptives long‐term.^41^ This practice, arising from an ethics of care framework, shows how parental responsibility can be fostered by showing interest in people’s lives, struggles, wishes and needs.

While occasionally the two frameworks seem to apply better to different kinds of cases, they can be complementary. For example, Feng and colleagues showed how the four principles helped them to analyse and understand conflicts within care, while they considered a relational approach preferable in order to determine how to support families.^42^ Earlier, we cited Lambert, Scheiner and Campbell, who weighed the various principles and concluded that the principle of justice should be decisive for making ethically good decisions.^43^ While this starting point roots them in the ethical framework of weighing interests, the way they defined justice showed a compassionate approach to the situation of the parents and the injustices they faced in a broader social context, and an understanding of how these factors challenged access to care for themselves and their children. So, these authors combined the two ethical frameworks.

The framework of weighing conflicting interests mostly focuses on ‘whether or not to intervene’, whereas the ethics of care framework approach is about ‘how to intervene’. The main question in the framework of conflicting interests is whether it is morally acceptable to intervene. Although there is also some 'how to intervene' in this framework, for example in Dondorp's and De Wert's claim that interventions should meet the criteria of effectiveness, proportionality, and subsidiarity. The framework of conflicting interests can make visible the dilemmas, whereas the framework of ethics of care can foster finding solutions, especially regarding how to foster supportive interaction with vulnerable families.^44^

These two frameworks can also help to provide more insights into two bottlenecks of care that we identified: collaboration and trust. We outlined earlier how the framework of competing interests can complicate collaboration between AOD and child welfare services. A strength of the ethics of care framework is that it can support this collaboration by outlining the shared interests between the services: supporting vulnerable families.

Another issue that professionals and parents identified is that there seems to be a certain mutual mistrust between parents and services: services frequently suspect that parents hide information, whereas parents are often afraid that they will be criticized about their parenting, their parental love, or, in the worst‐case scenario, even lose child custody. An ethics of care approach can help foster trust between the different parties involved (parents and various services). Care ethicist Tronto’s model of five stages of caring is particularly helpful in this regard.^45^ While the first four stages largely describe the process of caring, namely (1) caring about, (2) taking care of, (3) care giving, and (4) care receiving, the fifth stage of ‘caring with’ allows for a feedback loop with the other stages. After caring needs have been identified and responded to, and the care is being well received, it is necessary to return to the first stage, thereby continuously improving the quality of care. In this sense, care is an ever‐ongoing engagement. This cycle of care can result in more trust between struggling families and various services, because in this fifth stage care becomes a shared responsibility. A good example in this regard is the AOD service in which we recruited parents. This agency hires former patients who are now recovered as experiential treatment experts. And indeed, the parents we interviewed said that they found it very helpful to talk to someone who knew what they were going through. We could also see this attitude of caring in various child welfare workers, who stated how important it was to listen to the children and identify their needs. Although in other parts of interviews some child welfare workers also used a strong rhetoric of competing interests, ‘we defend the children’, they also used elements of the ethics of care framework. The ethics of care framework can support services and families in becoming aware of how to create more mutual trust.

## 4 CONCLUSION

We have sketched two ethical frameworks to use in designing care for families in which there is parental substance dependency: a framework of weighing conflicting interests according to the principles of autonomy, beneficence, non‐maleficence, and justice; and an ethics of care framework which focuses on the relationships and contexts in which people make decisions.

We argued that the framework of ethics of care is underused and hardly visible in the literature on parental substance dependency. If we focus solely on universal principles, many contextual and relational factors that are crucial for providing care for these families are omitted from the picture. A greater awareness of an ethics of care framework in addition to the weighing of conflicting interests framework is required. This ethics of care framework is already implicitly emerging from interviews with parents and professionals and in the literature on perinatal substance dependency. However, children, as well as the unborn, can benefit from an ethics of care approach.

Explicating these frameworks can support professionals in their practice, and also in their collaboration with services and parents. An increased awareness of which framework one is using and why could help in the identification of shared values that could strengthen collaboration between services, parents and professionals.

## Footnotes

^1^

Barnard, M., & McKeganey, N. (2004). The impact of parental problem drug use on children: What is the problem and what can be done to help? *Society for the Study of Addiction*, *99*(5), 552–559.10.1111/j.1360-0443.2003.00664.x15078229

^2^

Garcia, S. A. (1993). Maternal drug abuse: Laws and ethics as agents of just balances and therapeutic interventions. *Substance Use and Misuse*, *28*(13), 1311–1339; Taylor, A., & Kroll, B. (2004). Working with parental substance misuse: Dilemmas for practice. *British Journal of Social Work*, *34*(8), 1115–1132.10.3109/108260893090621908294173

^3^

Kalichman, S. C. (1999). Mandated reporting as an ethical dilemma. In S.C. Kalichman (Ed.), *Mandated reporting of suspected child abuse: Ethics, law, and policy* (pp. 43–63). Washington, DC: American Psychological Association; Alvarez, K. M., Kenny, M. C., Donohue, B., & Carpin, K. M. (2004). Why are professionals failing to initiate mandated reports of child maltreatment, and are there any empirically based training programs to assist professionals in the reporting process? *Aggression and Violent Behavior*, *9*(5), 563–578; Snoek, A., Dijkstra, B. A. G., Markus, W., Van der Meer‐Jansma, M., De Wert, G., & Horstkötter, D. (2021). “I wish I had help earlier. We could have been happier sooner.” Bystander effects in the care for alcohol‐dependent parents. *Frontiers in Psychology*, forthcoming. 10.3389/fpsyg.2021.656320 PMC828292434276478

^4^

Taylor & Kroll, op. cit. note 2; Drabble, L. (2007). Pathways to collaboration: Exploring values and collaborative practice between child welfare and substance abuse treatment fields. *Child Maltreatment*, *12*(1), 31–42; Niccols, A., Milligan, K., Smith, A., Sword, W., Thabane, L., & Henderson, J. (2012). Integrated programs for mothers with substance abuse issues and their children: A systematic review of studies reporting on child outcomes. *Child Abuse and Neglect*, *36*(4), 308–322.

^5^

Taylor & Kroll, op. cit. note 2.

^6^

Drabble, op. cit. note 4; He, A. S., Traube, D. E., & Young, N. K. (2014). Perceptions of parental substance use disorders in cross‐system collaboration among child welfare, alcohol and other drugs, and dependency court organizations. *Child Abuse and Neglect*, *38*(5), 939–951.10.1016/j.chiabu.2013.10.02024268380

^7^

Ibid; Young, N. K., & Gardner, S. (2002). *Navigating the pathways: Lessons and promising practices in linking alcohol and drug services with child welfare*. Rockville, MD: National Clearinghouse for Alcohol and Drug Information; Hunter, T. (2003). Child welfare and alcohol and other drug treatment (AOD): Bridging the gap to comprehensive services. *Journal of Family Social Work, 74*(4), 63–73.

^8^

Drabble, op. cit. note 4, p. 32.

^9^

Greipp, M. (1997). Ethical decision making and mandatory reporting in cases of suspected child abuse. *Journal of Pediatric Healthcare*, *11*(6), 258–265.10.1016/s0891-5245(97)90081-x9423410

^10^

Harwin, J., & Forrester, D. (2002). *Parental substance misuse and child welfare: A study of social work with families in which parents misuse drugs or alcohol*. London, UK: Nuffield Foundation, First stage report for the Nuffield Foundation, p.41.

^11^

Berends, L. (2011). Embracing the visual: Using timelines with in‐depth interviews on substance use and treatment. *Qualitative Report*, *16*(1), 1–9.

^12^

Abma, T. A., Baur, V. E., Molewijk, B., & Widdershoven, G. A. (2010). Inter‐ethics: Towards an interactive and interdependent bioethics. *Bioethics*, *24*(5), 242–255.10.1111/j.1467-8519.2010.01810.x20500761

^13^

We present more empirical findings in another paper Snoek et al., op cit. note 3.

^14^

Dondorp, W., & De Wert, G. (2017). Prenatal child protection. Ethics of pressure and coercion in prenatal care for addicted pregnant women. In K. Hens, D. Cutas & D. Horstkötter (Eds.), *Parental responsibility in the context of neuroscience and genetics* (p.121–131). New York, NY: Springer; Feng, J. Y., Chen, Y. W., Fetzer, S., Feng, M. C., & Lin, C. L. (2012). Ethical and legal challenges of mandated child abuse reporters. *Children and Youth Services Review*, *34*(1), 276–280; Lambert, B., Scheiner, M., & Campbell, D. (2010). Ethical issues and addiction. *Journal of Addictive Diseases*, *29*(2), 164–174.

^15^

Beauchamp, T., & Childress, J. F. (2002). *Principles of biomedical ethics* (5th ed.). *Erlanger Medical Ethics Orientation Manual*. Oxford, U.K.: Oxford University Press.

^16^

Dondorp & De Wert, op. cit. note 14; Lambert et al., op. cit. note 14.

^17^

Feng et al., op. cit. note 14.

^18^

Dondorp & De Wert, op. cit. note 14.

^19^

Ibid: 165.

^20^

Hart, C. L. (2013). *High price: A neuroscientist’s journey of self‐discovery that challenges everything you know about drugs and society*. New York, NY: HarperCollins.

^21^

Lambert et al. op. cit. note 14, p. 173.

^22^

Greipp, op. cit. note 9.

^23^

Ibid: 263.

^24^

Kalichmann, op. cit. note 3.

^25^

Watson, H., & Levine, M. (1988). Psychotherapy and mandated reporting of child abuse. *Amer. J*. *Orthopsychiat*.*, 59*(2), 246–256.10.1111/j.1939-0025.1989.tb01656.x2712158

^26^

Feng et al., op. cit. note 14.

^27^

Garcia, op. cit. note 2.

^28^

Ibid.

^29^

Benoit, C., Stengel, C., Marcellus, L., Hallgrimsdottir, H., Anderson, J., Mackinnon, K., … Charbonneau, S. (2013). Providers’ constructions of pregnant and early parenting women who use substances. *From Health* *Behaviours* *to Health Practices: Critical Perspectives*, *36*(2), 252–263.10.1111/1467-9566.1210624528306

^30^

Myers, J. E. B. (1991). A limited role for the legal system in responding to maternal substance use during pregnancy. *Notre Dame Journal of Law, Ethics & Public Policy*, *5*(3), 747–782.11651057

^31^

Gilligan, C. (2014). In a different voice: Women’s conceptions of self and morality. In D. T. Meyers (Ed.), *Feminist social thought: A reader* (pp. 481–517). Cambridge, MA: Harvard University Press; Tronto, J. C. (1993). *Moral boundaries: A political argument for an ethic of care*. New York, NY: Routledge.

^32^

Blacksher, E. (2002). On being poor and feeling poor: Low socioeconomic status and the moral self. *Theoretical Medicine and Bioethics*, *23*(6), 455–470; Jessup, M. A., Humphreys, J. C., Brindis, C. D., & Lee, K. A. (2003). Extrinsic barriers to substance abuse treatment among pregnant drug dependent women. *Journal of Drug Issues*, *33*(2), 285–304; Matthews, S., Dwyer, R., & Snoek, A. (2017). Stigma and self‐stigma in addiction. *Journal of Bioethical Inquiry*, *14*(2), 275–286.

^33^

Barnes, V. (2007). Young people’s views of children’s rights and advocacy services: A case for ‘caring’ advocacy? *Child Abuse Review*, *16*, 140–152; Holland, S. (2010). Looked after children and the ethic of care. *British Journal of Social Work*, *40*(6), 1664–1680; Marcellus, L. (2004). Feminist ethics must inform practice: Interventions with perinatal substance users. *Health Care for Women International*, *25*(8), 730–742.10.1080/0739933049047558415371078

^34^

Söderström, K., & Skolbekken, J. A. (2012). Pregnancy and substance use – The Norwegian § 10‐3 solution. *NAD Publication*, *29*(2), 155–171.

^35^

Dondorp & De Wert, op. cit. note 14.

^36^

Niccols et al., op. cit. note 4.

^37^

Feng et al., op. cit. note 14; Garcia, op. cit. note 2; Marcellus, op. cit. note 33; Söderström & Skolbekken, op. cit. note 34.

^38^

Garcia, op. cit. note 2.

^39^

Feng et al., op. cit. note 14.

^40^

Rijlaarsdam, C. W., Van Sambeek, D., & Rust, L. (2019). Reproductive and contraceptive healthcare for vulnerable people in the Netherlands: Pregnant not now! *European Journal of Public Health*, *29*(4), 209.

^41^

Ibid.

^42^

Feng et al., op cit. note 4.

^43^

Lambert et al., op. cit. note 4.

^44^

We would like to thank the anonymous reviewer for suggesting this specification to us.

^45^

Tronto, J. (2013). *Caring democracy: Markets, equality and justice*. New York, NY: New York University Press.

